# Pain Management After Surgery: A Brief Review

**DOI:** 10.5812/kowsar.22287523.3443

**Published:** 2012-01-01

**Authors:** Saeed Shoar, Sara Esmaeili, Saeid Safari

**Affiliations:** 1 Student Scientific Research Center (SSRC), Tehran University of Medical Sciences (TUMS), Tehran, Iran; 2 Department of Anesthesiology, Tehran University of Medical Sciences (TUMS), Tehran, Iran

**Keywords:** Analgesia, Pain, Postoperative Period

## Abstract

Proper pain management, particularly postoperative pain management, is a major concern for clinicians as well as for patients undergoing surgery. Although many advances have been made in the field of pain management, particularly during the past decades, not all patients achieve complete relief from postoperative pain. In this paper, we have emphasized the importance of postoperative analgesia and discussed the new developments in this field.

## 1. History

Proper pain management, particularly postoperative pain management, is a major concern for clinicians as well as for patients undergoing surgery. Patients commonly enquire about the level of pain they may experience after an operation (1). Postoperative pain not only affects the patients’ operative outcome, well being, and satisfaction from medical care, but also directly affects the development of tachycardia, hyperventilation, decrease in alveolar ventilation, transition to chronic pain, poor wound healing, and insomnia, which in turn may impact the operative outcomes (2-4). After drowsiness and digestive discomfort (i.e., nausea and vomiting), pain is most common cause of discharge delay in patients undergoing ambulatory surgery. The rapidly increasing number of complex surgical procedures that are performed in an outpatient setting has made perioperative and postoperative pain management very essential (1-3). 

Although many advances have been made in the field of pain management, particularly during the past decades, not all patients achieve complete relief from postoperative pain (3, 5, 6). The myriad aspects in which advances have been made in this field can be summarized as follows: recognizing the molecular target (peripheral or central) for blocking the pain signals, developing functional pharmaceuticals that affect the molecular target, determining the routes and modes of analgesic administration, and developing novel methods of analgesia (1).

Pain management is mainly classified on the basis of the use of pharmacological and nonpharmacological protocols; pharmacological protocols involve the use of opioid and nonopioid drugs, whereas nonpharmacological protocols involve the use of different routes of drug administration. 

## 2. Current Status

Postoperative pain management is an important but undervalued aspect of perioperative care. In the past decade, postoperative pain management, including the management of surgery-related and surgical pain, has been extensively studied (7). 

The nociceptive nature of postoperative pain (perception of pain after surgical insult) should be considered important in pain management because it may lead to conditions, such as hyperalgesia and allodynia, in which the central sensitivity to pain increases (8-10). Therefore, the central perception of pain should be studied along with the pathway via which pain signals are transmitted to the centrum.

The advances in the recognition of various targets for blocking pain signals have led to the development of an extensive list of protocols that combine the approved analgesic products, which have different mechanisms of action, with different methods of administration (11). However, the choice of an appropriate pain management protocol by pain care providers should be based on important factors such as the patients’ comorbidities, psychological conditions, and exposure to analgesics, as well as the surgical procedures performed and the operative site (1). The choice of an appropriate pain management protocol is very important in a multimodal pain care approach.

## 3. Management

The options for pain management are classified on the basis of the administration routes, mechanisms of action, and types of drugs. In the following sections, we have briefly described the above-mentioned classification criteria (1, 7, 11-13).

### 3.1. Administration Route

Oral, intravenous (IV), intramuscular, subcutaneous, rectal, transdermal, intrathecal, and epidural routes are the common routes of administration. Other promising options include neuronal blocks such as neuraxial blocks and peripheral nerve blocks. Some of the advanced techniques for pain management include epidural analgesia (which is efficacious but difficult to manage because it involves the administration of peripheral nerve blocks via catheters) and extended-duration analgesia (which can be administered at home).

### 3.2. Mechanism of Action

The agents used for pain management can be subdivided on the basis of their mechanisms of action into the following categories: analgesics (opioids and acetaminophen) or anti-inflammatory agents (nonsteroidal anti-inflammatory drugs [NSAIDs]). 

### 3.3. Types of Drugs

The different types of drugs include conventional drugs, e.g., acetaminophen (which is safe but its total dose needs to be carefully monitored), NSAIDs (which may reduce the opioid-related side effects), and opioids (which are the preferred drugs of choice); nontraditional drugs, e.g., ketamine (which is an excellent analgesic at very low doses), gabapentin (which is both an analgesic and anxiolytic agent); and intravenous patient-controlled drugs, e.g., morphine, fentanyl, hydromorphone, IV opioids, and meperidine.

## 4. Multimodal Analgesia

Although the use of combination therapy for pain management is relatively new, many different combinations of drugs are already available (14). As the number of patients undergoing minimally invasive surgeries continues to increase, adjunctive analgesics, including local and nonopioid analgesics, are being increasingly used for pain management; however, opioids are still very commonly used in the management of moderate to severe postoperative pains (15, 16). In fact, while a drug may have adverse effects at high doses, an adjunctive drug may reduce its adverse effects or intolerability (17-20). Recent evidence suggests that the reduction in these adverse effects may be best achieved by using a combination of protocols involving both central and peripheral-acting drugs and devices (12). 

## 5. Unresolved Issues

Despite the progress made in the field of postoperative pain management, it is important to conduct studies on modifying multimodal analgesic protocols for specific surgical procedures and on individual patients’ needs (11). Such studies would benefit anesthesiologists and pain care providers because the findings of these studies would lead to the development of an intelligent approach to customize specific multimodal protocols for pain management. Greater collaboration between medical experts is required to improve postoperative pain management in patients. Moreover, surgeons, anesthesiologists, nurses, physiotherapists, and other technicians involved in perioperative procedures and postoperative recovery should play an efficient role in pain management.

## 6. Recent Advances and New Horizons

Medical practitioners have become increasingly concerned about adequate pain management because of the increasing number of complex outpatient procedures and ambulatory surgeries. The advent of the newly introduced extended-action epidural morphine and iontophoretic transdermal fentanyl, which are particularly effective when used in combination with capsaicin, ketamine, gabapentin, pregabalin, dexmedetomidine, and tapentadol as adjunctives, has benefited the field of pain management. Moreover, the possibility of administering these products via the intranasal, regional, transdermal, and pulmonary routes has opened endless opportunities for research in the field of postoperative patient-controlled analgesia (PCA) (1).

From the patients’ point of view, efficient pain management depends mainly on the efforts of the health care providers to help the patients manage the pain. Therefore, it is important to consider the severity of pain experienced by the patients while deciding the best analgesic protocols for helping them tolerate the discomfort. 
